# Proenkephalin A and Heterogeneity of Hydrocortisone Treatment Effect in Septic Shock: A Post Hoc Analysis of the VANISH Trial

**DOI:** 10.1097/CCE.0000000000001496

**Published:** 2026-10-21

**Authors:** David B. Antcliffe, Florian Uhle, Dominic C. Marshall, Ashleigh D. Green, Anthony C. Gordon, Lui G. Forni

**Affiliations:** 1 Division of Anaesthetics, Pain Medicine and Intensive Care, Department of Surgery and Cancer, Imperial College London, London, United Kingdom.; 2 Intensive Care Unit, Charing Cross Hospital, Imperial College Healthcare NHS Trust, London, United Kingdom.; 3 Medical Department, SphingoTec GmbH, Hennigsdorf, Germany.; 4 School of Medicine, University of Surrey and Intensive Care Unit, Royal Surrey Hospital NHS Foundation Trust, Guildford, United Kingdom.

**Keywords:** corticosteroids, hydrocortisone, proenkephalin A, renal failure, sepsis, septic shock

## Abstract

**OBJECTIVES:**

Heterogeneity of treatment effect is thought to drive conflicting results for the benefits of corticosteroids in sepsis. Proenkephalin A (PENK) is a biomarker of renal function. Given the kidney’s role in sepsis pathophysiology and corticosteroid metabolism, we hypothesized that baseline renal function, assessed using PENK, would be associated with hydrocortisone efficacy.

**DESIGN:**

A post hoc analysis of a double-blind, randomized clinical trial in septic shock (Effect of Early Vasopressin vs. Norepinephrine on Kidney Failure in Patients with Septic Shock [VANISH]).

**SETTING:**

ICUs across the United Kingdom.

**PATIENTS:**

One hundred seventy-three patients admitted to ICU with septic shock.

**INTERVENTIONS:**

Norepinephrine vs. vasopressin and hydrocortisone vs. placebo.

**MEASUREMENTS AND MAIN RESULTS:**

PENK was measured in plasma, and patients were dichotomized using a PENK concentration above 89 pmol/L. PENK greater than or equal to 89 pmol/L was associated with 28-day mortality (odds ratio, 3.24; 95% CI, 1.62–6.74). We found no interaction between PENK and randomization to vasopressin or norepinephrine on outcome. However, there was a significant interaction between the PENK groups and hydrocortisone or placebo use in those patients who reached the maximum vasopressor threshold (*n* = 111) for kidney failure-free days (high PENK [≥ 89 pmol/L] placebo 26 [13–28], hydrocortisone 11 [0–26] and low PENK placebo 28 [15–28], hydrocortisone 28 [28–28]; *p* = 0.01). Patients with high PENK receiving hydrocortisone had the fewest kidney failure-free days. Although we found no significant interaction for 28-day mortality (*p* = 0.14), the patients with high PENK who received hydrocortisone had the highest mortality (high PENK hydrocortisone 50%, placebo 32% and low PENK hydrocortisone 14%, placebo 24%).

**CONCLUSIONS:**

This exploratory analysis identified a possible interaction between baseline PENK and hydrocortisone treatment on kidney failure-free days. These findings require validation in independent and prospective studies.

**KEY POINTS**

**Question**: Is baseline renal function, assessed using proenkephalin A (PENK), associated with clinical outcome and hydrocortisone efficacy?

**Findings**: Patients with septic shock who had high PENK levels had the highest 28-day mortality. Patients randomized to receive hydrocortisone in the Effect of Early Vasopressin vs. Norepinephrine on Kidney Failure in Patients with Septic Shock (VANISH) trial who met study criteria to receive it and had a high PENK had the fewest kidney failure-free days compared with all other groups (high PENK with placebo or low PENK with hydrocortisone or placebo). Although there was no significant interaction, 28-day mortality was also highest in high PENK patients receiving hydrocortisone.

**Meaning**: This exploratory analysis suggests that patients with septic shock and a high PENK level may have worse outcomes and generates the hypothesis that renal dysfunction may modify the effect of hydrocortisone, a finding that should be validated in other datasets and prospective studies.

Sepsis is a syndrome of acute organ dysfunction due to dysregulated response to infection (1). Acute kidney injury (AKI) occurs in 22–53% of patients with sepsis and is associated with high mortality rates (2).

Proenkephalin A 119–159 (PENK) is a stable byproduct of enkephalin synthesis, endogenous opioids whose receptors are highly expressed in the kidneys and are linked to kidney function (3). It is measurable in plasma and is an accurate surrogate for glomerular filtration rate (4) and an early and dynamic biomarker associated with AKI, need for renal replacement therapy, and mortality in patients with sepsis and other critical illnesses (5–10). It has potential advantages over other measures of renal function, such as creatinine or cystatin C, as it is unaffected by factors such as muscle mass and chronic inflammation, which can confound other measures.

Despite many trials, treatment for sepsis remains limited, with failure to find novel treatments being, to some extent, a result of the heterogeneity of this condition (11, 12). Finding ways to identify better which patients may respond to sepsis therapies is a research priority (13, 14). Improving our ability to identify which patients have underlying renal dysfunction could serve as one way to stratify patients for treatment. For example, previous studies have suggested that vasopressin may improve outcomes in patients with sepsis at risk of renal failure compared with norepinephrine (15) and that patients receiving vasopressin have lower use of renal replacement therapy than those receiving norepinephrine (16, 17). Similarly, corticosteroids have been studied for decades as adjunctive therapies in septic shock, yet large-scale trials have yielded conflicting results (18, 19). There is emerging evidence that this may be due to heterogeneity of treatment effect for corticosteroids (20–22). One of the first studies investigating the use of corticosteroids for septic shock found that those patients with renal failure at enrollment (defined as a creatinine > 176.8 μmol/L) had higher mortality if they received methylprednisolone (23). The association between corticosteroid use and mortality in patients with sepsis-associated AKI has also been suggested by subsequent retrospective analyses (24). However, this interaction has not been studied in subsequent corticosteroid trials (16, 18, 19, 25–29).

Given these findings and the kidney’s pivotal role in sepsis pathophysiology and corticosteroid metabolism, we hypothesized that baseline renal function, assessed using the novel kidney function biomarker PENK, would not only predict outcome in patients with septic shock but would predict response to vasopressin or hydrocortisone therapy.

Some of the preliminary findings of this study have been presented as an abstract (30).

## MATERIALS AND METHODS

### Datasets

Clinical observations and biomarker levels were taken from the Effect of Early Vasopressin vs. Norepinephrine on Kidney Failure in Patients With Septic Shock (VANISH) randomized controlled trial, which was a factorial (2 × 2) double-blind randomized trial conducted between 2013 and 2015. Inclusion criteria consisted of adult patients with sepsis requiring vasopressors. Patients were randomized to receive vasopressin or norepinephrine, with hydrocortisone or placebo. Hydrocortisone or placebo was only administered if the maximum infusion rate of the first study drug was required to achieve an adequate mean arterial pressure (0.06 U/min vasopressin or 12 µg/min norepinephrine) (16).

The original VANISH trial was provided with ethics approval from the Oxford A research ethics committee (ref 12/SC/0014, trial registration ISRCTN 20769191) and informed written consent was obtained from patients or their legal representatives. Ethical approval for the use of samples from the trial in the current study was provided by Imperial College Healthcare Tissue Bank, which is approved by Wales REC3 to release human material for research (22/WA/0214). Approval was granted in March 2024. All procedures were conducted in accordance with the Declaration of Helsinki.

### Blood Sample Collection

Plasma samples were collected upon recruitment and were available from 173 patients. Blood was collected into EDTA tubes and was separated locally (spun at 1000 g for 10 min), frozen according to standardized operating procedures, and then sent to the coordinating center in batches for storage at –80°C. Samples were sent to a central laboratory for PENK measurement using an in vitro diagnostic-certified chemiluminometric immunoassay (sphingotest penKid; SphingoTec GmbH, Hennigsdorf, Germany [31]) with assays conducted blinded to treatment allocation and outcome. A PENK concentration above the upper normal reference (≥ 89 pmol/L) was set as a cutoff to define abnormal kidney function (32), as defined in the assay’s instructions for diagnostic use.

### Outcomes

To confirm the prognostic benefit for PENK in this population as described in other populations, we used 28-day mortality as the primary outcome measure. For the assessment of heterogeneity of treatment effect, we used kidney failure-free days (i.e., the number of days alive and free of kidney failure), defined using the Acute Kidney Injury Network (AKIN) group stage 3 definition (33), during the 28 days after randomization, as used as the primary outcome in the original trial (**Supplementary Methods**, https://links.lww.com/CCX/B692) (16). This outcome was chosen as not only was it the primary outcome for the original trial, but as a composite outcome, which included the rate of kidney injury resolution and occurrence of new episodes of renal failure, it would be able to detect effects of the drugs if they either altered the rate of renal failure resolution or the incidence of new renal failure. The definition of kidney failure-free days used in the original trial contained no additional penalty for death, so in this analysis, to account for the worst outcome of death, which could compete with the number of kidney failure-free days, a sensitivity analysis was also performed where death was coded as –1 kidney failure-free days.

Secondary exploratory outcomes for assessment of heterogeneity of treatment effect were 28-day, ICU and hospital mortality, rates of kidney failure, weaning from vasopressors for greater than 24 hours, time to shock reversal, duration of mechanical ventilation, and mean total Sequential Organ Failure Assessment score.

Treatment effect was assessed on an intention-to-treat basis for the vasopressor allocation, but for the hydrocortisone vs. placebo analysis, where patients were only eligible to receive hydrocortisone/placebo if they reached the maximum infusion of the first study drug (16), only patients who received treatment as randomized were included for this comparison.

### Statistical Analysis

Association between PENK and 28-day mortality was evaluated using binary logistic regression. Unadjusted and adjusted for confounding variables identified using a directed acyclic graph (**Supplementary Fig. S1**, https://links.lww.com/CCX/B692) are reported.

For assessment of heterogeneity of treatment effect for kidney failure-free days, PENK status and drug interaction were explored using ordinal regression with adjustment for disease and shock severity (Supplementary Fig. S1, https://links.lww.com/CCX/B692). This analysis was used due to the ordinal nature of this outcome measure and its ability to handle skewed data and confounding variables. As missingness in the covariates (Acute Physiology and Chronic Health Evaluation [APACHE] II score and lactate) was low (< 3%), these were not imputed, and patients with missing data were excluded from adjusted analysis. Absolute differences in kidney failure-free days with a 95% CI were calculated using the Wilcoxon test. Secondary outcomes were summarized by PENK group and treatment allocation. PENK treatment interactions were tested using logistic regression for binary outcomes, linear regression for continuous outcomes, and Cox regression for time-to-event outcomes. In regression analysis, the group without renal failure and those receiving norepinephrine or placebo were used as the reference groups. Differences in survival were displayed using Kaplan-Meier curves, with log-rank tests for significance across all groups.

Because research blood samples were only available for a subset of 173 patients from the VANISH trial population, we conducted an exploratory analysis to explore the interaction between renal dysfunction and use of corticosteroids using clinical parameters available in the whole trial cohort. Two approaches were taken. First, patients were dichotomized into having renal failure or not based on the presence of AKIN stage 3 as described in the original trial article (16) or using a baseline creatinine cutoff of greater than 176.84 μmol/L as suggested previously to identify a group with higher mortality when receiving corticosteroids (see **Supplementary Fig. S2**, https://links.lww.com/CCX/B692, for a demonstration of these cohorts) (23). Interaction between renal failure groups and drug allocation was assessed for kidney failure-free days using ordinal regression, and 28-day mortality was analyzed using binary logistic regression and displayed with Kaplan-Meier curves.

Statistical analysis was carried out in R, Versions 4.3.2 and 4.6.0 (Vienna, Austria) (34), and *p* values of less than 0.05 denoted statistical significance; *p* values were not corrected for multiple testing, so the reported *p* values should be regarded as nominal and hypothesis-generating.

## RESULTS

Of the total trial population of 409 participants, 173 had samples available at baseline for measurement of PENK. Generally, sampled patients were similar to those who did not undergo sampling (**Supplementary Table S1**, https://links.lww.com/CCX/B692); for example, they had similar rates of acute renal failure, defined as AKI stage 3, at enrollment and low rates of renal replacement therapy. Of the sampled patients, 64% were male and had a median age of 65 and an APACHE II score of 23. Mortality at 28 days was 47 of 173 (27%). As expected, those patients from this cohort who had died by day 28 had evidence of more severe disease at baseline (**Supplementary Table S2**, https://links.lww.com/CCX/B692).

Blood samples were collected at randomization, which was a median of 0.25 hours (interquartile range [IQR], 0.08–0.75 hr) before initiation of randomized norepinephrine/vasopressin and 3 hours (IQR, 1–6 hr) before initiation of hydrocortisone/placebo. Sampled patients were dichotomized by baseline PENK measurement (≥ 89 pmol/L), with 82 of 173 (47%) being in the high PENK group. Patients who had high PENK were older and had features of more severe disease, including higher APACHE II scores, lactate, and creatinine. Patients who had high PENK were less likely to have an abdominal source of infection but more likely to have a source listed as “other,” which included urinary tract infections. This could also account for why fewer patients who had high PENK required mechanical ventilation than those who were not (**Table 1** and **Supplementary Table S3**, https://links.lww.com/CCX/B692). Similar trends were seen when patients were divided into PENK and drug combinations (**Supplementary Tables S4** and **S5**, https://links.lww.com/CCX/B692).

**TABLE 1.** Comparison of Patients Who Were Proenkephalin A Positive and Proenkephalin A Negative

Variable	High PENK	Low PENK
*N*	82	91
Men, *n*/total (%)	54/82 (66)	56/91 (62)
Age, median (IQR), yr	69 (59–79)	62 (47–75)
Acute Physiology and Chronic Health Evaluation II score, median (IQR)	26 (21–32)	22 (18–28)
Recent surgical history, *n*/total (%)	8/82 (10)	18/91 (20)
Preexisting conditions, *n*/total (%)		
Ischemic heart disease	16/82 (20)	12/91 (13)
Severe chronic obstructive pulmonary disease	6/82 (7)	4/91 (4)
Chronic kidney failure	6/82 (7)	2/91 (2)
Cirrhosis	5/82 (6)	6/91 (7)
Cancer	10/82 (12)	12/91 (13)
Immunocompromised	5/82 (6)	6/91 (7)
Diabetes	23/82 (28)	17/91 (19)
Source of infection, *n*/total (%)		
Lung	33/82 (40)	39/88 (44)
Abdomen	9/82 (11)	26/88 (30)
Soft tissue or line	3/82 (4)	3/88 (3)
Other	37/82 (45)	20/88 (23)
Organ failure, *n*/total (%)^a^		
Respiratory	29/82 (35)	33/89 (37)
Kidney	34/82 (41)	6/91 (7)
Liver	7/72 (10)	5/81 (6)
Hematological	5/81 (6)	5/87 (6)
Neurologic	23/80 (29)	32/86 (37)
Volume of IV fluid in previous 4 hr, median (IQR), mL	1260 (632–2123)	1019 (540–1691)
Time from onset of shock to receiving first study drug, median (IQR), hr	3.3 (2.0–5.2)	4.0 (2.0–5.0)
Patients receiving open-label vasopressor at randomization, *n*/total (%)	68/82 (83)	80/91 (88)
Norepinephrine dose at randomization, median (IQR), μg/kg/min (in those taking norepinephrine)	0.17 (0.10–0.34)	0.14 (0.08–0.21)
Mechanical ventilation, *n*/total (%)	36/82 (44)	55/91 (60)
Renal replacement therapy, *n*/total (%)	4/82 (5)	2/91 (2)
Randomized to vasopressin, *n*/total (%)	37/82 (45)	51/91 (56)
Randomized to hydrocortisone, *n*/total (%)	45/82 (55)	42/91 (46)
Receiving randomized hydrocortisone, *n*/total (%)	34/56 (61)	21/55 (38)
Day 28 mortality, *n*/total (%)	32/82 (39)	15/91 (16)
Kidney failure-free days, median (IQR)	21 (1–28)	28 (25–28)

IQR = interquartile range, PENK = proenkephalin A.

a Organ failure is defined as having a Sequential Organ Failure Assessment score of 3 or more except for acute renal failure which was defined as having acute kidney injury stage 3. Further details can be found in Supplementary Table S3 (https://links.lww.com/CCX/B692).

Comparison of patients who had high PENK (≥ 89 pmol/L) vs. those who had low PENK with data collected at the time of randomization.

### Association of PENK With Mortality

PENK showed only moderate correlation with serum creatinine (Spearman rho = 0.69; 95% CI, 0.61–0.76), suggesting that while there is overlap, a meaningful proportion of variation remains unshared. In this cohort of patients with septic shock high PENK was associated with increased 28-day mortality (odds ratio [OR], 3.24; 95% CI, 1.62–6.74; *p* = 0.001), mortality in high PENK (32/82 [39%]) and low PENK (15/91 [16%] with an absolute risk difference of 23% [95% CI, 10–36%]), consistent with findings from previous studies (5, 6, 8). To ensure this association was not confounded by disease severity, we adjusted for APACHE II score and lactate (Supplementary Fig. S1, https://links.lww.com/CCX/B692). In this adjusted analysis, the association between PENK status and mortality remained (OR, 2.23; 95% CI, 1.02–4.98; *p* = 0.045); this also remained the case if baseline creatinine and AKIN stage 3 renal failure were included as covariates (OR, 3.22; 95% CI, 1.25–8.56; *p* = 0.02), suggesting that PENK status confers additional risk information to conventional measures and definitions of renal dysfunction.

### Exploration of Heterogeneity of Treatment Effect

We found no interaction between PENK status and vasopressor assignment (*n* = 173) on the primary outcome of kidney failure-free days or any of the other outcomes of interest (**Supplementary Table S6**, https://links.lww.com/CCX/B692). Although 28-day mortality was different between groups, this was driven by the PENK status of the patient, with those who had high PENK having the highest mortality (**Figs. 1** and **2**) irrespective of treatment allocation.

**Figure 1.**
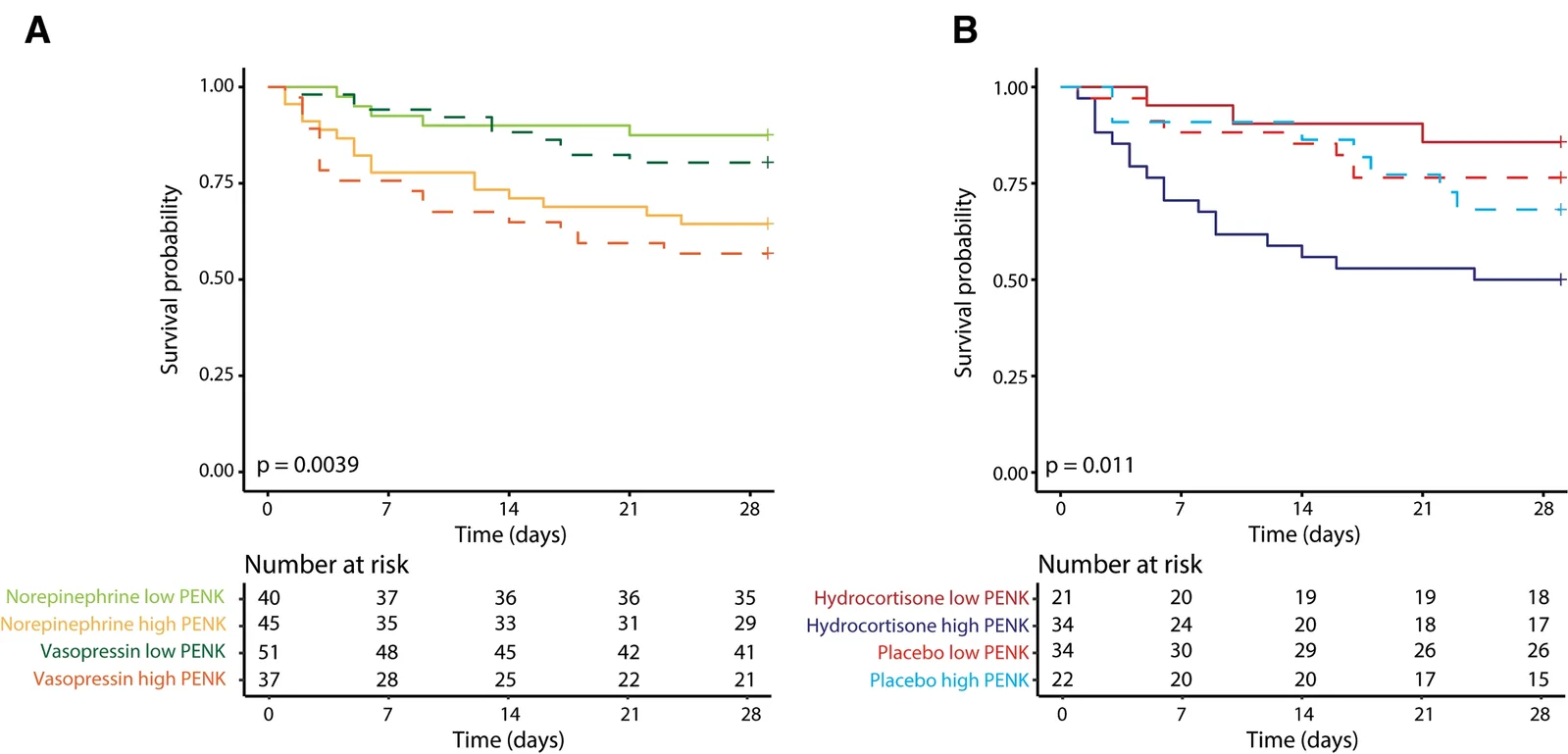
*Kaplan-Meier curves* showing 28-d survival stratified by (**A**) proenkephalin A (PENK) status and the first study drug (*n* = 173, low PENK norepinephrine—*solid pale green*, low PENK vasopressin—*dashed dark green*, high PENK norepinephrine—*solid pale orange*, and high PENK vasopressin—*dashed dark orange*) and (**B**) PENK status and the second study drug (*n* = 111, low PENK placebo—*dashed pale red*, low PENK hydrocortisone—*solid dark red*, high PENK placebo—*dashed pale blue*, and high PENK hydrocortisone—*solid dark blue*). *p* values are given by the log-rank test testing the difference in survival across all groups.

**Figure 2.**
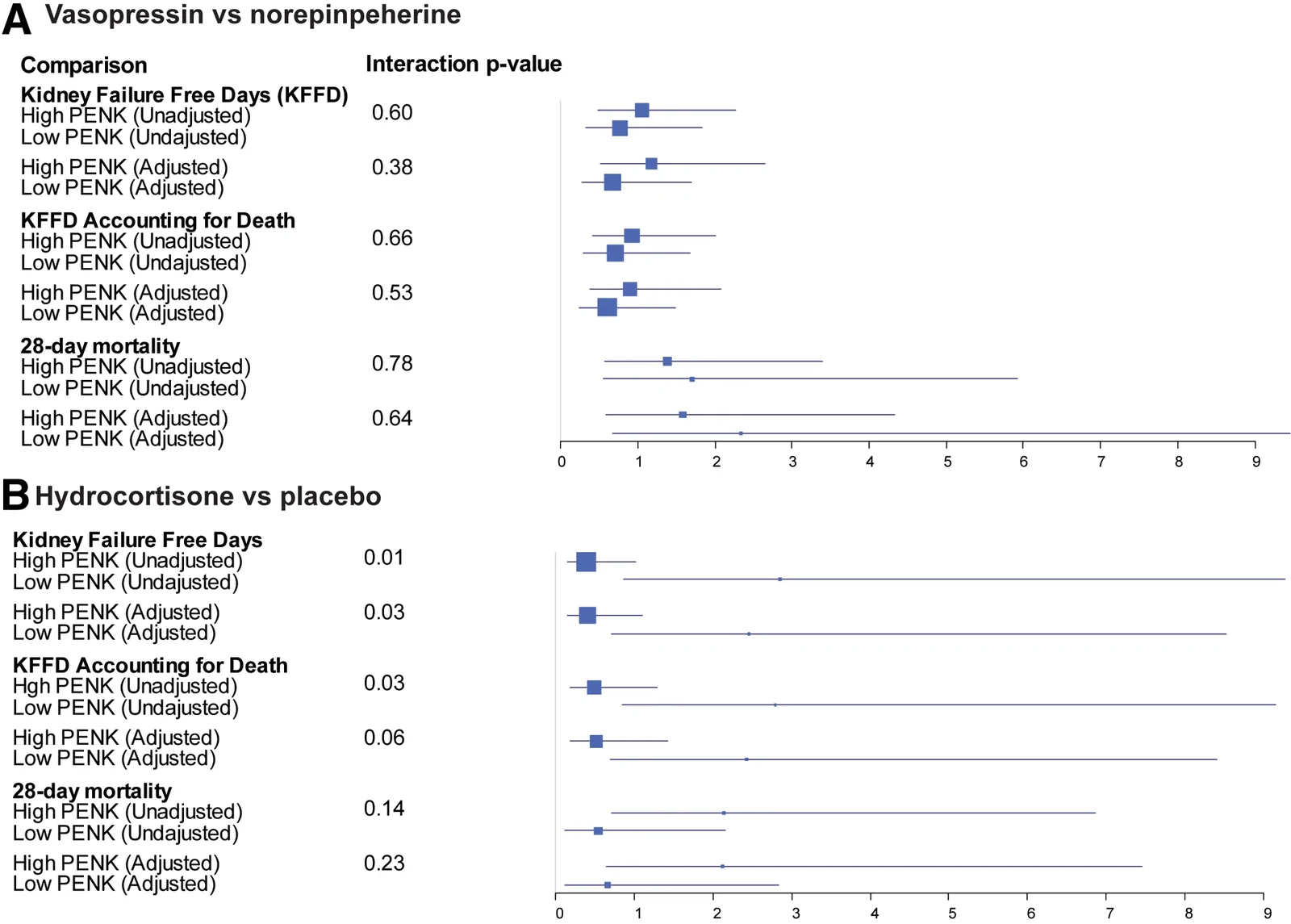
*Forest plots* comparing kidney failure-free days (KFFDs), KFFDs incorporating death, and day 28 mortality for vasopressin vs. norepinephrine (*n* = 173; **A**) and hydrocortisone vs. placebo (*n* = 111; **B**), this analysis is performed in only those patients who received the second study drug. *p* values are given for the interaction term for each analysis, and the adjusted analysis accounts for baseline Acute Physiology and Chronic Health Evaluation (APACHE) II score and lactate (In the vasopressin vs. norepinephrine analysis, APACHE II score was unavailable in one patient and lactate in four; in the hydrocortisone vs. placebo analysis, APACHE II score was unavailable in one and lactate in two patients; these patients were omitted from the adjusted analysis). PENK = proenkephalin A.

When patients who received the second study drug (hydrocortisone vs. placebo; *n* = 111/173) were considered, there was a significant interaction between PENK status and treatment allocation (*p* = 0.01) on the number of kidney failure-free days. Patients receiving hydrocortisone who had high PENK had the fewest kidney failure-free days (high PENK hydrocortisone median, 11 d; IQR, 0–26 d) compared with high PENK placebo 26 (IQR, 13–28; OR, 0.39; 95% CI, 0.15–1.01) and low PENK hydrocortisone 28 (IQR, 28–28), low PENK placebo 28 (15–28; OR, 2.85; 95% CI, 0.87–9.28; **Table 2**; Fig. 2). This equated to an absolute reduction in renal failure-free days of 3.5 days (95% CI, 0–15 d) in patients given hydrocortisone in those whose PENK was high and 0 days (95% CI, –2 to 0 d) in those whose PENK was low. These analyses also remained significant after correction for APACHE II score and lactate at randomization (*p* = 0.03). When the analysis was performed accounting for death as the worst outcome, the interaction between PENK status and drug remained significant in unadjusted analysis (*p* = 0.03) but became nonsignificant (*p* = 0.06) after adjustment (Table 2 and Fig. 2).

**TABLE 2.** Interaction Between Proenkephalin A Status and Drug Allocation on Kidney Failure-Free Days

Group	*n*	Kidney Failure-Free Days			Kidney Failure-Free Days With Death Coded As –1		
		Median (IQR)	OR (95% CI)	Adjusted OR (95% CI)	Median (IQR)	OR (95% CI)	Adjusted OR (95% CI)
Interaction	—	—	0.14 (0.03–0.63); *p* = 0.01	0.16 (0.03–0.82); *p* = 0.03	—	0.17 (0.04–0.82); *p* = 0.03	0.21 (0.04–1.05); *p* = 0.06
Placebo low PENK	34	28 (15–28)	Reference	Reference	28 (6–28)	Reference	Reference
Hydrocortisone low PENK	21	28 (28–28)	2.85 (0.87–9.28)	2.46 (0.71–8.53)	28 (28–28)	2.79 (0.85–9.16)	2.43 (0.70–8.41)
Placebo high PENK	22	26 (13–28)	Reference	Reference	26 (–1 to 28)	Reference	Reference
Hydrocortisone high PENK	34	11 (0–26)	0.39 (0.15–1.01)	0.40 (0.15–1.11)	–1 (–1 to 26)	0.49 (0.18–1.29)	0.51 (0.18–1.42)

IQR = interquartile range, PENK = proenkephalin A, OR = odds ratio.

Comparison of kidney failure-free days (median [IQR]) between patients who had high PENK (≥ 89 pmol/L) or low PENK given either hydrocortisone or placebo. ORs with 95% CIs were calculated using ordinal regression both in an unadjusted model and a model adjusting for Acute Physiology and Chronic Health Evaluation (APACHE) II score and lactate at randomization. In the regression analysis, the reference groups were those who were low PENK and those receiving placebo. *p* values are given for the interaction terms (two patients had lactate and one APACHE II unavailable, so were not included in the adjusted analysis).

The interaction between PENK status and treatment allocation, hydrocortisone or placebo, for 28-day mortality was nonsignificant in unadjusted (*p* = 0.14) or adjusted analysis (*p* = 0.23). However, across all groups the high PENK patients receiving hydrocortisone had the highest 28-day mortality (high PENK hydrocortisone, 17/34 [50%]); high PENK placebo (7/22 [32%] for an absolute risk difference of 18% [95% CI, –8% to 44%]); low PENK hydrocortisone (3/21 [14%]); low PENK placebo (8/34 [24%] for an absolute risk difference of –10% [95% CI, –30% to 11%]; Figs 1 and 2; **Supplementary Table S7**, https://links.lww.com/CCX/B692). We found no significant interactions between hydrocortisone/placebo allocation and PENK status on any of the secondary outcomes (Supplementary Table S7, https://links.lww.com/CCX/B692).

As we had dichotomized PENK as a cutoff for defining AKI, we conducted exploratory analyses dividing PENK into quartiles and as a continuous variable (**Supplementary Table S8** and **Fig. S4**, https://links.lww.com/CCX/B692). These data were directionally consistent with the prespecified cutoff, but more work in a larger sample size will be needed to establish the ideal cutoff to identify the optimum cutoff for treatment-effect modification.

To further explore the possible association between renal function at trial enrollment and the effect of hydrocortisone, we conducted an exploratory analysis of the whole trial population who received either hydrocortisone or placebo (*n* = 293) based on the presence of AKI defined as having AKIN stage 3 or more or a creatinine greater than 176.8 μmol/L as previously described (**Supplementary Fig. S3**, https://links.lww.com/CCX/B692, shows the overlap of cohorts) (23). Defining acute renal failure with either AKIN stage 3 or a creatinine greater than 176.8 μmol/L showed a similar direction of effect for the interaction of kidney failure and hydrocortisone use for kidney failure-free days; however, these did not reach statistical significance (**Table 3**). In both cases, the number of kidney failure-free days was lowest in the renal failure hydrocortisone group (Table 3). In neither case was there an interaction between kidney failure and drug for 28-day mortality (**Supplementary Table S9**, https://links.lww.com/CCX/B692), although the absolute number of deaths was highest in patients with baseline renal failure who received hydrocortisone across all definitions (**Fig. 3** and Supplementary Table S9, https://links.lww.com/CCX/B692). An exploratory analysis exploring creatinine as quartiles and continuous variables is presented in Supplementary Table S8 and Figure S4 (https://links.lww.com/CCX/B692).

**TABLE 3.** Interaction Between Acute Kidney Injury Status and Drug Allocation on Kidney Failure-Free Days

Group	*n*	Kidney Failure-Free Days			Kidney Failure-Free Days With Death Coded As –1		
		Median (IQR)	OR (95% CI)	Adjusted OR (95% CI)	Median (IQR)	OR (95% CI)	Adjusted OR (95% CI)
Acute Kidney Injury Network stage 3							
Interaction	—	—	0.73 (0.27–1.92); *p* = 0.52	0.60 (0.22–1.67); *p* = 0.33	—	0.71 (0.27–1.89); *p* = 0.49	0.56 (0.20–1.60); *p* = 0.28
Placebo no AKI	116	28 (13–28)	Reference	Reference	28 (–1 to 28)	Reference	Reference
Hydrocortisone no AKI	111	28 (11–28)	0.99 (0.60–1.62)	1.00 (0.60–1.67)	28 (–1 to 28)	1.00 (0.61–1.66)	1.06 (0.62–1.79)
Placebo AKI	31	17 (0–25)	Reference	Reference	17 (–1 to 25)	Reference	Reference
Hydrocortisone AKI	35	7 (0–25)	0.72 (0.31–1.66)	0.60 (0.25–1.46)	–1 (–1 to 25)	0.72 (0.31–1.66)	0.59 (0.24–1.47)
Creatinine threshold							
Interaction	—	—	0.55 (0.23–1.31); *p* = 0.18	0.46 (0.19–1.16); *p* = 0.10	—	0.60 (0.24–1.45); *p* = 0.25	0.51 (0.20–1.30); *p* = 0.16
Placebo no AKI	102	28 (14–28)	Reference	Reference	28 (–1 to 28)	Reference	Reference
Hydrocortisone no AKI	92	28 (13–28)	1.20 (0.70–2.07)	1.22 (0.69–2.15)	28 (–1 to 28)	1.20 (0.69–2.07)	1.23 (0.69–2.20)
Placebo AKI	44	19 (1–26)	Reference	Reference	19 (–1 to 26)	Reference	Reference
Hydrocortisone AKI	54	14 (0–25)	0.66 (0.33–1.31)	0.57 (0.28–1.16)	1 (–1 to 25)	0.71 (0.35–1.43)	0.63 (0.30–1.31)

AKI = acute kidney injury, IQR = interquartile range, OR = odds ratio.

Comparison of kidney failure-free days in all patients and accounting for death as the worst outcome between hydrocortisone and placebo stratified by AKI status defined as Acute Kidney Injury Network (AKIN) stage 3 or a creatinine > 176.8 μmol/L. ORs with 95% CIs were calculated using ordinal regression both in an unadjusted model and a model adjusting for Acute Physiology and Chronic Health Evaluation (APACHE) II score and lactate at randomization. In the regression analysis, patients with no AKI and those receiving placebo were used as reference groups. *p* values are given for the interaction terms (One patient had no baseline creatinine but an AKIN stage 3 classification. One patient had no APACHE score, and six had no lactate, so were not included in adjusted analyses).

**Figure 3.**
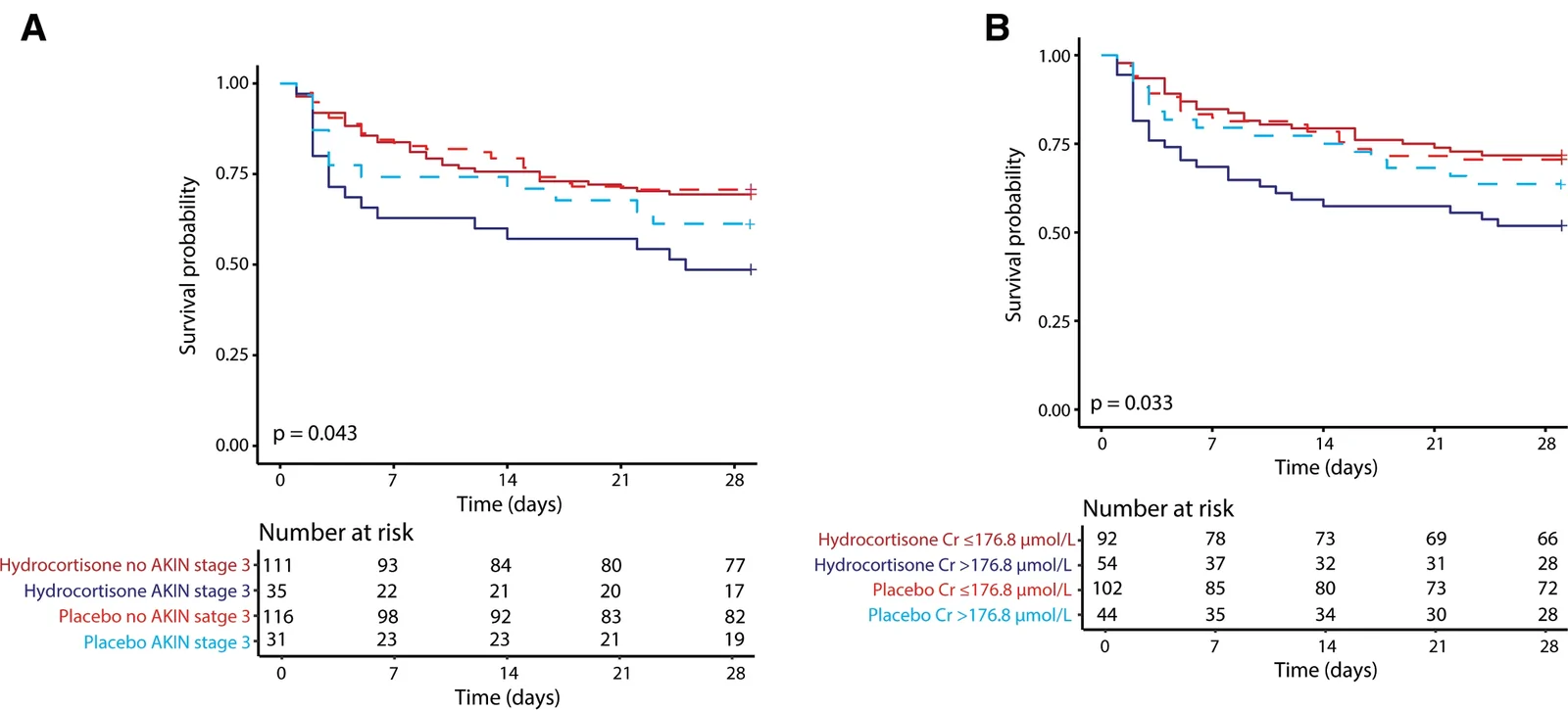
*Kaplan-Meier curves* showing 28-d survival. **A**, Kidney failure defined as patients with Acute Kidney Injury Network (AKIN) stage 3 renal failure. **B**, Kidney failure defined as a baseline creatinine (Cr) greater than 176.8 μmol/L. On the *Kaplan-Meier curves*: no acute kidney injury (AKI) placebo—*dashed pale red*, no AKI hydrocortisone—*solid dark red*, AKI placebo—*dashed pale blue*, and AKI hydrocortisone—*solid dark blue; p* values are given for the log-rank test testing the difference in survival across all groups.

The lack of significant interaction in the models using more conventional ways to define renal dysfunction suggested that PENK may identify differences in treatment effect that these measures are unable to. As an exploratory analysis, we repeated our adjusted models including baseline creatinine and baseline renal failure as defined by AKIN stage 3 along with APACHE II score and lactate as covariates to assess if the interaction remained when conventional measures of renal function were accounted for. The interaction for kidney failure-free days remained significant (*p* = 0.04) with the same directions of effect as in our primary analysis (OR hydrocortisone vs. placebo in low PENK 2.11 [0.60–7.36] and OR hydrocortisone vs. placebo in high PENK 0.36 [0.12–1.06]). Similar results were seen when death was accounted for in the definition of kidney failure-free data, although the *p* value for interaction was outside of the significance threshold (*p* = 0.08; OR hydrocortisone vs. placebo in low PENK 2.08 [0.60–7.24] and OR hydrocortisone vs. placebo in high PENK 0.48 [0.16–1.44]).

## DISCUSSION

In this hypothesis-generating study, we demonstrated that in a cohort of patients admitted to the ICU with septic shock, a plasma PENK greater than 89 pmol/L identified patients with higher mortality, consistent with the previous literature (5, 6, 8, 10). Importantly, when patients were dichotomized by plasma PENK at trial enrollment, patients who had high PENK and received hydrocortisone had the fewest kidney failure-free days. Although we also found a similar direction of effect using a creatinine threshold of 176.8 μmol/L or AKIN stage 3 to define AKI, neither reached statistical significance. This supports the need for prospective validation in a larger dataset where more patients can have PENK measurements to understand if PENK is able to identify differential treatment effects not identified by conventional renal biomarkers and definitions.

The finding that PENK was associated with worse outcomes in septic shock is consistent with previously reported studies (5, 6, 8, 10). PENK has been shown to predict patients who will develop AKI (5, 6, 9, 35) after admission. However, sepsis and, more generally, critical illness are littered with biomarkers that have some prognostic value, although clinical utility is often not addressed or is unknown. This result is particularly interesting because of the finding that high PENK was associated with fewer kidney failure-free days in those patients who received hydrocortisone, with a signal that mortality may be highest in this group, although with no statistically significant interaction between PENK status and drug. Of note, this finding is consistent with the trial by Bone et al (23) in which patients with a creatinine greater than 176.8 μmol/L had increased mortality when receiving methylprednisolone. Despite this signal in early trials, more recent trials have not explored the association between AKI and response to corticosteroids in sepsis (16, 18, 19, 25–29), and there are few human studies looking at the effect of corticosteroids on sepsis-associated kidney injury. One recent retrospective analysis of large ICU databases suggested that glucocorticoid treatment was associated with an increased risk of ICU and hospital mortality when given to patients with sepsis-associated kidney injury defined using the Kidney Disease: Improving Global Outcomes (KDIGO) criteria (24). However, this association has not been consistently found, with a single center observational study of patients with sepsis-associated KDIGO-defined AKI suggesting an improvement in mortality with corticosteroids (36). Although our dataset is small, it generates the hypothesis that renal dysfunction may modify the effect of renal dysfunction, a hypothesis which could be tested in other datasets.

Despite these potential signals for harm, it is unclear what the underlying mechanism could be. Although several putative mechanisms may be proposed, none are measured in this study and remain unproven, and all would need further investigation. Potential mechanisms could include alterations in regional blood flow (37) induced by corticosteroid-related effects on vascular reactivity, low cortisol-binding proteins (38), and illness-related alterations of cortisol metabolism that might increase exposure to biologically active hydrocortisone in certain tissues. However, although potential mechanisms can be hypothesized, none can be proven in these data, and none explain why PENK may provide potential benefits over other measures of renal dysfunction.

Although the mechanisms are unclear, the current study adds to the growing evidence that there may be heterogeneity of treatment effect for corticosteroids given in critical illness. Subgroups of patients defined by gene expression (20, 22), circulating inflammatory mediators (21), or renal function (23, 24) have demonstrated harm when given corticosteroids. This supports the need for future prospective stratified trials that aim to confirm which patients benefit from corticosteroids and which are harmed, so that we can identify individuals who may benefit from this treatment.

Despite the strengths of this study, including the use of randomized trial data to evaluate heterogeneity of treatment effect and the use of predefined PENK thresholds, there are a number of limitations. First, the sample size of patients with PENK measurements is small. It represents a post hoc analysis of only the subset of patients who underwent blood sampling and, for the hydrocortisone vs. placebo analysis, those who met the eligibility criteria to receive the drug. The limited sample size and the number of outcomes assessed increase the risk of the results representing chance findings and type 1 error, and as the study was not powered to identify treatment-effect heterogeneity, there is also a risk of false negative findings. These limitations mean that the results should be viewed as exploratory and hypothesis-generating and will need to be validated in other cohorts and in prospective studies.

The primary outcome used in this analysis of kidney failure-free days is not entirely patient-centered and may not be the best to determine heterogeneity of treatment effect in future studies. Mortality may be a better outcome for future investigation; however, in this analysis, this was a secondary endpoint and did not reach statistical significance for an interaction, which may represent the small sample size. Finally, it remains unclear whether PENK itself, beyond being a surrogate of kidney dysfunction, is important in understanding heterogeneity of treatment effect for hydrocortisone in septic shock. However, our adjusted analysis supports that it may provide additional information to conventional measures and definitions of renal dysfunction such as creatinine and AKIN staging.

Finally, although the data used in this analysis are taken from a randomized trial, randomization occurred at study entry, and only a proportion of patients met the threshold to receive the hydrocortisone/placebo, the cohort that our analysis was restricted to. In this situation, it is possible that some of the benefits of randomization in terms of group balance no longer hold; however, as study drugs were based on randomized allocation and not clinician judgment, this still provides advantages over studies that have explored corticosteroid heterogeneity of treatment effect in observational data (21, 22) where confounding by indication cannot be excluded, and the use of blinding means that clinical decisions that could influence outcomes were not based on knowledge of treatment allocation. As we were aware of this limitation, we also performed an adjusted analysis that demonstrated the same direction of effect as the primary unadjusted analysis.

In conclusion, we have shown in a secondary analysis of a clinical trial that PENK is predictive of 28-day mortality and generated the hypothesis that high PENK may modify the effect of hydrocortisone, a finding that warrants evaluation in other datasets but does not yet establish the clinical role for PENK.

## Supplementary Material
