# Supplementary material for: Proenkephalin A and Heterogeneity of Hydrocortisone Treatment Effect in Septic Shock: A Post Hoc Analysis of the VANISH Trial
Source: Crit Care Explor. 2026 Oct 21;8(10):e1496. doi: 10.1097/CCE.0000000000001496 (PMC13686750; doi:10.1097/CCE.0000000000001496)

Proenkephalin A and Heterogeneity of Hydrocortisone Treatment Effect in Septic Shock:

A Post Hoc Analysis of the VANISH Trial – supplementary material.

David B. Antcliffe, Florian Uhle, Dominic C. Marshall, Ashleigh D. Green, Anthony C. Gordon, Lui G. Forni

Supplementary methods

Supplementary Table S1: Comparison of patients who did and did not have samples collected

Supplementary Table S2: Comparison of survivors and non-survivors who underwent sampling for PENK measurement.

Supplementary table S3: Comparison of clinical features of patients who had high or low PENK

Supplementary table S4: Comparison of clinical features of patients who had high or low PENK by randomization to vasopressin or norepinephrine.

Supplementary table S5: Comparison of clinical features of patients who had high or low PENK by placebo or hydrocortisone use.

Supplementary table S6: Comparison of outcomes of patients who had high or low PENK who were randomized to receive either vasopressin or norepinephrine.

Supplementary table S7: Comparison of outcomes of patients who had high or low PENK who were randomized to receive either placebo or hydrocortisone.

Supplementary table S8: Assessment of quartiles of PENK and creatinine and their relationship with kidney failure free days and 28-day mortality.

Supplementary table S9: Comparison of 28-day mortality rates between hydrocortisone and placebo stratified by kidney injury status defined by Acute Kidney Injury Network stage 3 or creatinine threshold.

Supplementary Figure S1: Directed acyclic graph showing potential sources of confounding

Supplementary Figure S2: Fow diagram showing the cohorts of patients used for each analysis

Supplementary Figure S3: Venn diagram showing overlap of patient cohorts

Supplementary Figure S4: Association of PENK and creatinine as continuous variables with kidney failure free days and mortality.

Supplementary Methods

Kidney failure free days

The number of kidney failure free days in the first 28 days was calculated as follows. Patients were scored as being alive or dead on each calendar day after randomization, and if they were alive they were scored as either having or being free from kidney failure using the daily data. Kidney failure was defined as having Acute Kidney Injury stage 3. In subjects who were discharged alive from ICU before day 28, the need for ongoing dialysis (RRT) treatment was collected post ICU discharge. (In this definition, subjects who died at some point in the 28 days may still have been scored as having organ failure free days while they were alive. Hence on this measure a subject who died may have more organ failure free days than one who did not.)

Kidney failure was defined by the Acute Kidney Injury Network (AKIN) group stage 3 definition (Mehta et al., 2007):

• increase in serum creatinine to >300% (>3-fold) from baseline

• or serum creatinine ≥354 μmol/L with an acute rise of at least 44 μmol/L

• or initiation of renal replacement therapy (for acute kidney injury)

• or a urine output of < 0.3 mL/kg/h for ≥24 hours

• or anuria for ≥12 hours

Supplementary Table S1: Comparison of patients who did and did not have samples available.

Variable	Sampled	Not Sampled
n	173	236
Men, No./total (%)	110/173 (64)	128/236 (54)
Age median (IQR), y	65 (53-77)	66 (57-76)
Caucasian ethnicity, No./total (%)	143/173 (83)	206/236 (87)
Weight, median (IQR), kg	75 (62-90)	74 (63-85)
BMI, median (IQR)	26 (22-31)	25 (22-29)
APACHE II score, median (IQR)	23 (19-30)	24 (19-30)
Recent surgical history, No./total (%)	26/173 (15)	47/236 (20)
Pre-existing conditions, No./total (%)		
Ischemic heart disease	28/173 (16)	34/236 (14)
Severe COPD	10/173 (6)	5/236 (2)
Chronic kidney failure	8/173 (5)	19/236 (8)
Cirrhosis	11/173 (6)	4/236 (2)
Cancer	22/173 (13)	25/236 (11)
Immunocompromised	11/173 (6)	17/236 (7)
Diabetes	40/173 (23)	50/236 (21)
Source of infection, No./total (%)		
Lung	72/170 (42)	93/230 (40)
Abdomen	35/170 (21)	58/230 (25)
Soft tissue or line	6/170 (4)	13/230 (6)
Other	57/170 (34)	66/230 (29)
Physiological variables, median (IQR)		
Mean arterial pressure, mm Hg	69 (62-76)	70 (62-78)
Heart rate, beats/min	94 (82-110)	100 (86-110)
Lactate, mmol/L	2.4 (1.5-4.0)	2.3 (1.3-4.0)
PaO2/FiO2, kPa	26 (17-41)	24 (15-38)
Creatinine, µmol/L	119 (78-198)	126 (73-213)
Bilirubin, µmol/L	15 (9-30)	15 (8-26)
Platelets, $\times 10^3/\mu L$	187 (118-285)	188 (126-291)
GCS	14 (5-15)	14 (3-15)
Organ failure, No./total (%)*		
Respiratory	62/171 (36)	87/231 (38)
Kidney	40/173 (23)	45/235 (19)
Liver	12/153 (8)	8/226 (4)
Hematological	10/168 (6)	12/230 (5)
Neurological	55/166 (33)	73/219 (33)
Volume of IV fluid in previous 4 h, median (IQR), mL	1098 (542-2000)	1166 (725-2141)
Time from onset of shock to receiving first study drug, median (IQR), h	3.7 (2.0-5.2)	3.3 (1.3-5.3)
Patients receiving open-label vasopressor at randomization, No./total (%)	148/173 (86)	200/236 (85)
Norepinephrine dose at randomization, median (IQR), µg/kg/min (in those taking norepinephrine)	0.15 (0.09-0.26)	0.19 (0.12-0.36)
Norepinephrine dose at randomization, median (IQR), µg/kg/min (in all patients)	0.13 (0.06-0.23)	0.15 (0.05-0.30)
Mechanical ventilation, No./total (%)	91/173 (53)	145/236 (61)
Renal replacement therapy, No./total (%)	6/173 (3)	5/236 (2)
Day-28 mortality No./total (%)	47/173 (27)	72/235 (31)

(Abbreviations: APACHE, Acute Physiology and Chronic Health Evaluation (range 0-72, a higher score corresponds to more severe illness and a higher risk of death); BMI, body mass index (calculated as weight in kilograms divided by height in meters squared); COPD, chronic obstructive pulmonary disease; GCS, Glasgow Coma Score (range 3-15, a lower score corresponds to a greater depression of consciousness); IQR, interquartile range; IV, intravenous; PaO_2/FiO_2, arterial oxygen partial pressure to fractional inspired oxygen.) * Organ failure is defined as having a Sequential Organ Failure Assessment (SOFA) score of 3 or more except for acute renal failure which was defined as having acute kidney injury stage 3.

Supplementary Table S2: Comparison of survivors and non-survivors who underwent sampling for PENK measurement.

**Variable**	**Survivors**	**Non-survivors**
n	126	47
Men, No./total (%)	82/126 (65)	28/47 (60)
Age median (IQR), y	65 (51-75)	67 (58-79)
Caucasian ethnicity, No./total (%)	103/126 (82)	40/47 (85)
Weight, median (IQR), kg	75 (62-90)	73 (61-94)
BMI, median (IQR)	26 (23-31)	28 (22-32)
APACHE II score, median (IQR)	23 (18-28)	28 (23-35)
Recent surgical history, No./total (%)	21/126 (17)	5/47 (11)
Pre-existing conditions, No./total (%)		
Ischemic heart disease	18/126 (14)	10/47 (21)
Severe COPD	8/126 (6)	2/47 (4)
Chronic kidney failure	6/126 (5)	2/47 (4)
Cirrhosis	7/126 (6)	4/47 (9)
Cancer	15/126 (12)	7/47 (15)
Immunocompromised	6/126 (5)	5/47 (11)
Diabetes	33/126 (26)	7/47 (15)
Source of infection, No./total (%)		
Lung	47/124 (38)	25/46 (54)
Abdomen	29/124 (23)	6/46 (13)
Soft tissue or line	6/124 (5)	0/46 (0)
Other	42/124 (34)	15/46 (33)
Physiological variables, median (IQR)		
Mean arterial pressure, mm Hg	69 (62-75)	69 (62-81)
Heart rate, beats/min	93 (82-110)	95 (84-112)
Lactate, mmol/L	2.2 (1.4-3.5)	2.8 (1.8-5.8)
PaO2/FiO2, kPa	32 (20-43)	21 (13-29)
Creatinine, µmol/L	112 (71-178)	163 (86-270)
Bilirubin, µmol/L	14 (9-27)	17 (9-44)
Platelets, $\times 10^3$/µL	179 (118-263)	199 (98-332)
GCS	14 (8-15)	10 (3-14)
Organ failure, No./total (%)*		
Respiratory	37/124 (30)	25/47 (53)
Kidney	21/126 (17)	19/47 (40)
Liver	8/108 (7)	4/45 (9)
Hematological	7/121 (6)	3/47 (6)
Neurological	34/121 (28)	21/45 (47)
Volume of IV fluid in previous 4 h, median (IQR), mL	1079 (588-1949)	1253 (500-2040)
Time from onset of shock to receiving first study drug, median (IQR), h	4.0 (2.0-5.1)	3.5 (2.0-5.3)
Patients receiving open-label vasopressor at randomization, No./total (%)	108/126 (86)	40/47 (85)
Norepinephrine dose at randomization, median (IQR), µg/kg/min (in those taking norepinephrine)	0.13 (0.08-0.22)	0.19 (0.14-0.33)
Norepinephrine dose at randomization, median (IQR), µg/kg/min (in all patients)	0.11 (0.05-0.20)	0.18 (0.09-0.32)
Mechanical ventilation, No./total (%)	63/126 (50)	28/47 (60)
Renal replacement therapy, No./total (%)	2/126 (2)	4/47 (9)

(Abbreviations: APACHE, Acute Physiology and Chronic Health Evaluation (range 0-72, a higher score corresponds to more severe illness and a higher risk of death); BMI, body mass index (calculated as weight in kilograms divided by height in meters squared); COPD, chronic obstructive pulmonary disease; GCS, Glasgow Coma Score (range 3-15, a lower score corresponds to a greater depression of consciousness); IQR, interquartile range; IV, intravenous; PaO_2/FiO_2, arterial oxygen partial pressure to fractional inspired oxygen.) *Organ failure is defined as having a Sequential Organ Failure Assessment (SOFA) score of 3 or more except for acute renal failure which was defined as having acute kidney injury stage 3.

Supplementary Table S3: Comparison of clinical features of patients who had either high or low PENK

Variable	High PENK	Low PENK
n	82	91
Men, No./total (%)	54/82 (66)	56/91 (62)
Age median (IQR), y	69 (59-79)	62 (47-75)
Caucasian ethnicity, No./total (%)	71/82 (87)	72/91 (79)
Weight, median (IQR), kg	80 (64-95)	75 (60-85)
BMI, median (IQR)	28 (23-33)	25 (22-31)
APACHE II score, median (IQR)	26 (21-32)	22 (18-28)
Recent surgical history, No./total (%)	8/82 (10)	18/91 (20)
Pre-existing conditions, No./total (%)		
Ischemic heart disease	16/82 (20)	12/91 (13)
Severe COPD	6/82 (7)	4/91 (4)
Chronic kidney failure	6/82 (7)	2/91 (2)
Cirrhosis	5/82 (6)	6/91 (7)
Cancer	10/82 (12)	12/91 (13)
Immunocompromised	5/82 (6)	6/91 (7)
Diabetes	23/82 (28)	17/91 (19)
Source of infection, No./total (%)		
Lung	33/82 (40)	39/88 (44)
Abdomen	9/82 (11)	26/88 (30)
Soft tissue or line	3/82 (4)	3/88 (3)
Other	37/82 (45)	20/88 (23)
Physiological variables, median (IQR)		
Mean arterial pressure, mm Hg	69 (62-78)	69 (62-75)
Heart rate, beats/min	92 (83-107)	95 (82-112)
Lactate, mmol/L	2.8 (1.8-4.7)	1.9 (1.4-3.4)
PaO_2/FiO_2, kPa	27 (17-40)	25 (18-42)
Creatinine, µmol/L	188 (123-331)	83 (63-121)
Creatinine >176.8 µmol/L No./total (%)	43/82 (52)	6/91 (7)
Bilirubin, µmol/L	14 (7-35)	16 (11-29)
Platelets, $\times 10^3$/µL	191 (122-278)	179 (104-292)
GCS	14 (6-15)	14 (3-15)
Organ failure, No./total (%)*		
Respiratory	29/82 (35)	33/89 (37)
Kidney	34/82 (41)	6/91 (7)
Liver	7/72 (10)	5/81 (6)
Hematological	5/81 (6)	5/87 (6)
Neurological	23/80 (29)	32/86 (37)
Volume of IV fluid in previous 4 h, median (IQR), mL	1260 (632-2123)	1019 (540-1691)
Time from onset of shock to receiving first study drug, median (IQR), h	3.3 (2-5.3.0)	4.0 (2.0-5.0)
Patients receiving open-label vasopressor at randomization, No./total (%)	68/82 (83)	80/91 (88)
Norepinephrine dose at randomization, median (IQR), µg/kg/min (in those taking norepinephrine)	0.17 (0.10-0.34)	0.14 (0.08-0.21)
Norepinephrine dose at randomization, median (IQR), µg/kg/min (in all patients)	0.14 (0.05-0.28)	0.12 (0.06-0.19)
Mechanical ventilation, No./total (%)	36/82 (44)	55/91 (60)
Renal replacement therapy, No./total (%)	4/82 (5)	2/91 (2)
Randomized to vasopressin No./total (%)	37/82 (45)	51/91 (56)
Randomized to hydrocortisone No./total (%)	45/82 (55)	42/91 (46)
Receiving randomized hydrocortisone No./total (%)	34/56 (61)	21/55 (38)
Day-28 mortality No./total (%)	32/82 (39)	15/91 (16)

(Abbreviations: APACHE, Acute Physiology and Chronic Health Evaluation (range 0-72, a higher score corresponds to more severe illness and a higher risk of death); BMI, body mass index (calculated as weight in kilograms divided by height in meters squared); COPD, chronic obstructive pulmonary disease; GCS, Glasgow Coma Score (range 3-15, a lower score corresponds to a greater depression of consciousness); IQR, interquartile range; IV, intravenous; PaO_2/FiO_2, arterial oxygen partial pressure to fractional inspired oxygen.) *Organ failure is defined as having a Sequential Organ Failure Assessment (SOFA) score of 3 or more except for acute renal failure which was defined as having acute kidney injury stage 3.

Supplementary Table S4: Comparison of patients randomized to norepinephrine or vasopressin by PENK status

Variable	Low PENK		High PENK	
	Norepinephrine	Vasopressin	Norepinephrine	Vasopressin
n	40	51	45	37
Men, No./total (%)	26/40 (65)	30/51 (59)	31/45 (69)	23/37 (62)
Age median (IQR), y	62 (45-77)	62 (47-71)	65 (57-78)	72 (64-80)
Caucasian ethnicity, No./total (%)	31/40 (78)	41/51 (80)	40/45 (89)	31/37 (84)
Weight, median (IQR), kg	75 (61-85)	73 (60-85)	80 (63-99)	80 (64-93)
BMI, median (IQR)	26 (22-31)	25 (22-31)	28 (22-31)	27 (23-34)
APACHE II score, median (IQR)	22 (16-27)	22 (19-28)	26 (20-32)	25 (21-32)
Recent surgical history, No./total (%)	8/40 (20)	10/51 (20)	5/45 (11)	3/37 (8)
Pre-existing conditions, No./total (%)				
Ischemic heart disease	6/40 (15)	6/51 (12)	7/45 (16)	9/37 (24)
Severe COPD	2/40 (5)	2/51 (4)	4/45 (9)	2/37 (5)
Chronic kidney failure	0/40 (0)	2/51 (4)	2/45 (4)	4/37 (11)
Cirrhosis	2/40 (5)	4/51 (8)	2/45 (4)	3/37 (8)
Cancer	6/40 (15)	6/51 (12)	5/45 (11)	5/37 (14)
Immunocompromised	2/40 (5)	4/51 (8)	3/45 (7)	2/37 (5)
Diabetes	9/40 (23)	8/51 (16)	14/45 (31)	9/37 (24)
Source of infection, No./total (%)				
Lung	15/38 (39)	24/50 (48)	19/45 (42)	14/37 (38)
Abdomen	16/38 (42)	10/50 (20)	5/45 (11)	4/37 (11)
Soft tissue or line	2/38 (5)	1/50 (2)	1/45 (2)	2/37 (5)
Other	5/38 (13)	15/50 (30)	20/45 (44)	17/37 (46)
Physiological variables, median (IQR)				
Mean arterial pressure, mm Hg	68 (57-75)	72 (63-76)	69 (63-78)	69 (62-75)
Heart rate, beats/min	95 (81-112)	99 (82-112)	92 (82-111)	94 (84-103)
Lactate, mmol/L	2.35 (1.55-3.43)	1.70 (1.30-3.45)	2.55 (1.40-5.13)	2.80 (1.93-4.70)
PaO_2/FiO_2, kPa	22 (18-42)	28 (16-43)	26 (18-36)	29 (17-44)
Creatinine, µmol/L	74 (56-131)	87 (70-119)	176 (125-320)	188 (116-337)
Bilirubin, µmol/L	14 (9-40)	18 (11-28)	14 (7-21)	13 (9-51)
Platelets, $\times 10^3/\mu L$	202 (131-293)	152 (101-278)	191 (121-266)	197 (125-326)
GCS	14 (3-15)	14 (3-15)	14 (3-15)	13 (8-15)
Organ failure, No./total (%)*				
Respiratory	15/40 (38)	18/49 (37)	19/45 (42)	10/37 (27)
Kidney	3/40 (8)	3/51 (6)	19/45 (42)	15/37 (41)
Liver	4/34 (12)	1/47 (2)	3/40 (8)	4/32 (13)
Hematological	2/36 (6)	3/51 (6)	3/45 (7)	2/36 (6)
Neurological	12/39 (31)	20/47 (43)	13/44 (30)	10/36 (28)
Volume of IV fluid in previous 4 h, median (IQR), mL	921 (411-1865)	1050 (547-1653)	1058 (407-2063)	1260 (911-2233)
Time from onset of shock to receiving first study drug, median (IQR), h	4.0 (2.7-4.9)	4.0 (2.0-5.3)	3.0 (2.0-5.4)	3.5 (2.0-5.2)
Patients receiving open-label vasopressor at randomization, No./total (%)	32/40 (80)	48/51 (94)	37/45 (82)	31/37 (84)
Norepinephrine dose at randomization, median (IQR), µg/kg/min (in those taking norepinephrine)	0.13 (0.08-0.19)	0.14 (0.09-0.24)	0.18 (0.10-0.37)	0.15 (0.09-0.34)
Norepinephrine dose at randomization, median (IQR), µg/kg/min (in all patients)	0.10 (0.03-0.16)	0.14 (0.08-0.23)	0.16 (0.06-0.30)	0.13 (0.05-0.26)
Mechanical ventilation, No./total (%)	24/40 (60)	31/51 (61)	24/45 (53)	12/37 (32)
Renal replacement therapy, No./total (%)	1/40 (3)	1/51 (2)	2/45 (4)	2/37 (5)

(Abbreviations: APACHE, Acute Physiology and Chronic Health Evaluation (range 0-72, a higher score corresponds to more severe illness and a higher risk of death); BMI, body mass index (calculated as weight in kilograms divided by height in meters squared); COPD, chronic obstructive pulmonary disease; GCS, Glasgow Coma Score (range 3-15, a lower score corresponds to a greater depression of consciousness); IQR, interquartile range; IV, intravenous; PaO_2/FiO_2, arterial oxygen partial pressure to fractional inspired oxygen.) *Organ failure is defined as having a Sequential Organ Failure Assessment (SOFA) score of 3 or more except for acute renal failure which was defined as having acute kidney injury stage 3.

Supplementary Table S5: Comparison of patients receiving randomized hydrocortisone or placebo by PENK status

	Low PENK		High PENK	
**Variable**	**Placebo**	**Hydrocortisone**	**Placebo**	**Hydrocortisone**
n	34	21	22	34
Men, No./total (%)	22/34 (65)	10/21 (48)	14/22 (64)	25/34 (74)
Age median (IQR), y	60 (45-76)	65 (46-78)	74 (64-79)	66 (57-79)
Caucasian ethnicity, No./total (%)	25/34 (74)	18/21 (86)	19/22 (86)	30/34 (88)
Weight, median (IQR), kg	74 (60-80)	73 (59-88)	71 (61-98)	85 (65-98)
BMI, median (IQR)	25 (21-30)	26 (23-32)	26 (23-34)	28 (23-31)
APACHE II score, median (IQR)	23 (20-30)	22 (19-30)	24 (19-32)	28 (22-36)
Recent surgical history, No./total (%)	8/34 (24)	6/21 (29)	4/22 (18)	1/34 (3)
Pre-existing conditions, No./total (%)				
Ischemic heart disease	0/34 (0)	5/21 (24)	5/22 (23)	7/34 (21)
Severe COPD	2/34 (6)	0/21 (0)	0/22 (0)	2/34 (6)
Chronic kidney failure	1/34 (3)	0/21 (0)	1/22 (5)	2/34 (6)
Cirrhosis	2/34 (6)	1/21 (5)	2/22 (9)	3/34 (9)
Cancer	6/34 (18)	4/21 (19)	2/22 (9)	3/34 (9)
Immunocompromised	3/34 (9)	2/21 (10)	1/22 (5)	2/34 (6)
Diabetes	4/34 (12)	7/21 (33)	7/22 (32)	6/34 (18)
Source of infection, No./total (%)				
Lung	18/34 (53)	8/19 (42)	6/22 (27)	19/34 (56)
Abdomen	8/34 (24)	7/19 (37)	4/22 (18)	2/34 (6)
Soft tissue or line	1/34 (3)	1/19 (5)	2/22 (9)	1/34 (3)
Other	7/34 (21)	3/19 (16)	10/22 (45)	12/34 (35)
Physiological variables, median (IQR)				
Mean arterial pressure, mm Hg	65 (59-74)	67 (57-75)	69 (64-70)	69 (59-78)
Heart rate, beats/min	101 (82-118)	101 (84-114)	94 (84-107)	92 (82-102)
Lactate, mmol/L	2.00 (1.45-4.35)	1.80 (1.25-2.85)	2.80 (1.88-4.70)	3.0 (1.9-5.1)
PaO_2/FiO_2, kPa	21 (16-37)	23 (19-41)	21 (12-40)	26 (20-37)
Creatinine, µmol/L	106 (65-127)	81 (57-118)	176 (89-278)	247 (149-364)
Bilirubin, µmol/L	17 (10-28)	15 (10-23)	12 (6-48)	13 (9-31)
Platelets, $\times 10^3/\mu L$	186 (102-273)	211 (95-310)	186 (133-349)	207 (122-267)
GCS	13 (3-15)	14 (3-15)	14 (7-15)	13 (3-15)
Organ failure, No./total (%)*				
Respiratory	18/33 (55)	8/20 (40)	7/22 (32)	15/34 (44)
Kidney	3/34 (9)	0/21 (0)	8/22 (36)	16/34 (47)
Liver	1/30 (3)	1/20 (5)	1/18 (6)	3/33 (9)
Hematological	2/32 (6)	2/21 (10)	1/21 (5)	3/34 (9)
Neurological	14/32 (44)	8/19 (42)	5/21 (24)	12/34 (35)
Volume of IV fluid in previous 4 h, median (IQR), mL	1250 (745-2020)	1067 (660-1900)	1207 (583-2538)	1257 (498-1790)
Time from onset of shock to receiving first study drug, median (IQR), h	3.5 (2.0-5.5)	4.4 (2.4-4.9)	3.3 (2.1-5.3)	3.3 (1.7-5.0)
Patients receiving open-label vasopressor at randomization, No./total (%)	30/34 (88)	18/21 (86)	18/22 (82)	28/34 (82)
Norepinephrine dose at randomization, median (IQR), µg/kg/min (in those taking norepinephrine)	0.17 (0.1-0.26)	0.15 (0.1-0.29)	0.21 (0.09-0.32)	0.23 (0.14-0.46)
Norepinephrine dose at randomization, median (IQR), µg/kg/min (in all patients)	0.13 (0.06-0.25)	0.14 (0.08-0.22)	0.14 (0.03-0.29)	0.17 (0.08-0.43)
Mechanical ventilation, No./total (%)	25/34 (74)	13/21 (62)	10/22 (45)	17/34 (50)
Renal replacement therapy, No./total (%)	1/34 (3)	0/21 (0)	1/22 (5)	2/34 (6)
Randomized vasopressin No./total (%)	23/34 (68)	7/21 (33)	12/22 (55)	16/34 (47)

(Abbreviations: APACHE, Acute Physiology and Chronic Health Evaluation (range 0-72, a higher score corresponds to more severe illness and a higher risk of death); BMI, body mass index (calculated as weight in kilograms divided by height in meters squared); COPD, chronic obstructive pulmonary disease; GCS, Glasgow Coma Score (range 3-15, a lower score corresponds to a greater depression of consciousness); IQR, interquartile range; IV, intravenous; PaO_2/FiO_2, arterial oxygen partial pressure to fractional inspired oxygen.) *Organ failure is defined as having a Sequential Organ Failure Assessment (SOFA) score of 3 or more except for acute renal failure which was defined as having acute kidney injury stage 3.

Supplementary table S6: Comparison of outcomes of patients who were had high (≥89 pmol/L) or low PENK randomized to either norepinephrine or vasopressin. P-values are for unadjusted interaction analysis.

Outcome	High PENK		Low PENK		p-value
	**Norepinephrine**	**Vasopressin**	**Norepinephrine**	**Vasopressin**	**-**
n	45	37	40	51	-
Kidney failure free days, median (IQR)	21 (1-28)	20 (1-28)	28 (26-28)	28 (25-28)	0.60‡
Kidney failure free days with death as the worst outcome, median (IQR)	21 (-1-28)	20 (-1-28)	28 (26-28)	28 (25-28)	0.66‡
Kidney failure, No./total (%)	32/45 (71)	23/37 (62)	11/40 (28)	13/51 (25)	0.65†
Day-28 mortality No./total (%)	16/45 (36)	16/37 (43)	5/40 (13)	10/51 (20)	0.78†
Hospital mortality No./total (%)	16/45 (36)	15/37 (41)	5/40 (13)	9/51 (18)	0.80†
ICU mortality No./total (%)	14/45 (31)	12/37 (32)	5/40 (13)	7/51 (14)	0.95†
No. weaned from vasopressors for >24 h, No./total (%)	38/45 (84)	34/37 (92)	37/40 (93)	49/51 (96)	0.97†
Time to shock reversal, median (IQR), hours	46 (18-75)	47 (28-90)	42 (23-90)	37 (16-70)	0.42€
Duration of mechanical ventilation, median (IQR), days	6 (2-14)	5 (2-10)	5 (2-18)	5 (2-9)	0.58‡
Mean total SOFA score, median (IQR)	6.0 (3.9-8.9)	5.6 (3.8-8.7)	4.4 (3.4-7.3)	3.9 (2.7-5.6)	0.37§

† Binary logistic regression, ‡ordinal regression, €Cox regression, §linear regression, applying a square root transform to the outcome

Supplementary table S7: Comparison of secondary outcomes of patients who had high PENK (≥89 pmol/L) or low PENK given either hydrocortisone or placebo. P-values are for unadjusted interaction analysis.

	High PENK		Low PENK		
	**Placebo**	**Hydrocortisone**	**Placebo**	**Hydrocortisone**	**p-value**
n	22	34	34	21	-
Kidney failure, No./total (%)	14/22 (64)	25/34 (74)	13/34 (38)	3/21 (14)	0.06†
Day-28 mortality No./total (%)	7/22 (32)	17/34 (50)	8/34 (24)	3/21 (14)	0.14†
Hospital mortality No./total (%)	7/22 (32)	16/34 (47)	7/34 (21)	3/21 (14)	0.25†
ICU mortality No./total (%)	5/22 (23)	13/34 (38)	6/34 (18)	3/21 (14)	0.31†
No. weaned from vasopressors for >24 h, No./total (%)	20/22 (91)	30/34 (88)	31/34 (91)	20/21 (95)	0.53†
Time to shock reversal, median (IQR), hours	71 (30-127)	48 (29-83)	68 (31-114)	40 (23-72)	0.32€
Duration of mechanical ventilation, median (IQR), days	10 (3-15)	6 (2-13)	6 (3-13)	5 (2-14)	0.86‡
Mean total SOFA score, median (IQR)	6.3 (4.1-8.0)	6.7 (3.7-10.0)	4.6 (3.2-5.6)	4.0 (2.9-6.3)	0.75§

† Binary logistic regression, ‡ordinal regression, €Cox regression, §linear regression, applying a square root transform to the outcome

Supplementary table S8: Exploratory analysis of heterogeneity of treatment effect for hydrocortisone vs placebo splitting PENK (median 92.8pg/ml (IQR: 57.3-154.7)) or creatinine (128 μmol/L (IQR: 80-224)) into quartiles for kidney failure free days, kidney failure free days incorporating death as -1 and 28-day mortality in the population that received those drugs.

Kidney Failure Free Days (KFFD):

	**PENK**				**Creatinine**			
	**Hydrocortisone**		**Placebo**		**Hydrocortisone**		**Placebo**	
**Quartile**	n	KFFD (median (IQR)	n	KFFD (median (IQR)	n	KFFD (median (IQR)	n	KFFD (median (IQR)
**1**	10	28 (27-28)	17	28 (18-28)	33	28 (25-28)	39	28 (14-28)
**2**	11	28 (28-28)	17	26.0 (14-28)	35	28 (21-28)	38	28 (14-28)
**3**	14	11 (0-27)	14	18 (9-28)	33	13 (0-28)	41	25 (7-28)
**4**	20	12 (0-26)	8	26 (21-28)	45	15 (0-26)	28	17 (0-25)

Kidney Failure Free Days (KFFD) with death coded as -1:

	**PENK**				**Creatinine**			
	**Hydrocortisone**		**Placebo**		**Hydrocortisone**		**Placebo**	
**Quartile**	n	KFFD (median (IQR)	n	KFFD (median (IQR)	n	KFFD (median (IQR)	n	KFFD (median (IQR)
**1**	10	28 (27-28)	17	28 (10-28)	33	28 (25-28)	39	28 (-1 - 28)
**2**	11	28 (28-28)	17	26 (4-28)	35	28 (-1-28)	38	28 (-1 - 28)
**3**	14	-1 (-1 - 27)	14	12.5 (-1 - 28)	33	0 (-1 - 28)	41	25 (-1 - 28)
**4**	20	8 (-1 - 26)	8	26 (21 - 28)	45	2 (-1 - 26)	28	17 (-1 - 25)

28-day Mortality:

	**PENK**		**Creatinine**	
	**Hydrocortisone**	**Placebo**	**Hydrocortisone**	**Placebo**
**Quartile**	**Death/Total (%)**	**Death/Total (%)**	**Death/Total (%)**	**Death/Total (%)**
**1**	1/10 (10)	4/17 (24)	6/33 (18)	12/39 (31)
**2**	2/11 (18)	4/17 (24)	9/35 (26)	10/38 (26)
**3**	7/14 (50)	6/14 (43)	16/33 (48)	13/41 (32)
**4**	10/20 (50)	1/8 (13)	21/45 (47)	11/28 (39)

Supplementary table S9: Comparison of 28-day mortality between hydrocortisone and placebo stratified by acute kidney injury (AKI) status defined as Acute Kidney Injury Network (AKIN) stage 3 or creatinine 176.8 µmol/L as used previously (Bone et al., 1987). P-values are given for the unadjusted interaction term from binary logistic regression.

Group	AKIN stage 3		Creatinine threshold	
	**Deaths/total (%)**	**p-value**	**Deaths/total (%)**	**p-value**
Placebo No AKI	34/116 (29)	0.43	30/102 (29)	0.30
Placebo AKI	12/31 (39)		16/44 (36)	
Hydrocortisone No AKI	34/111 (31)		26/92 (28)	
Hydrocortisone AKI	18/35 (51)		26/54 (48)	

Supplementary Figure S1 Directed acyclic graph showing potential confounding variables. Data sources used to control for these in the models are shown with red text (AKI, acute kidney injury).

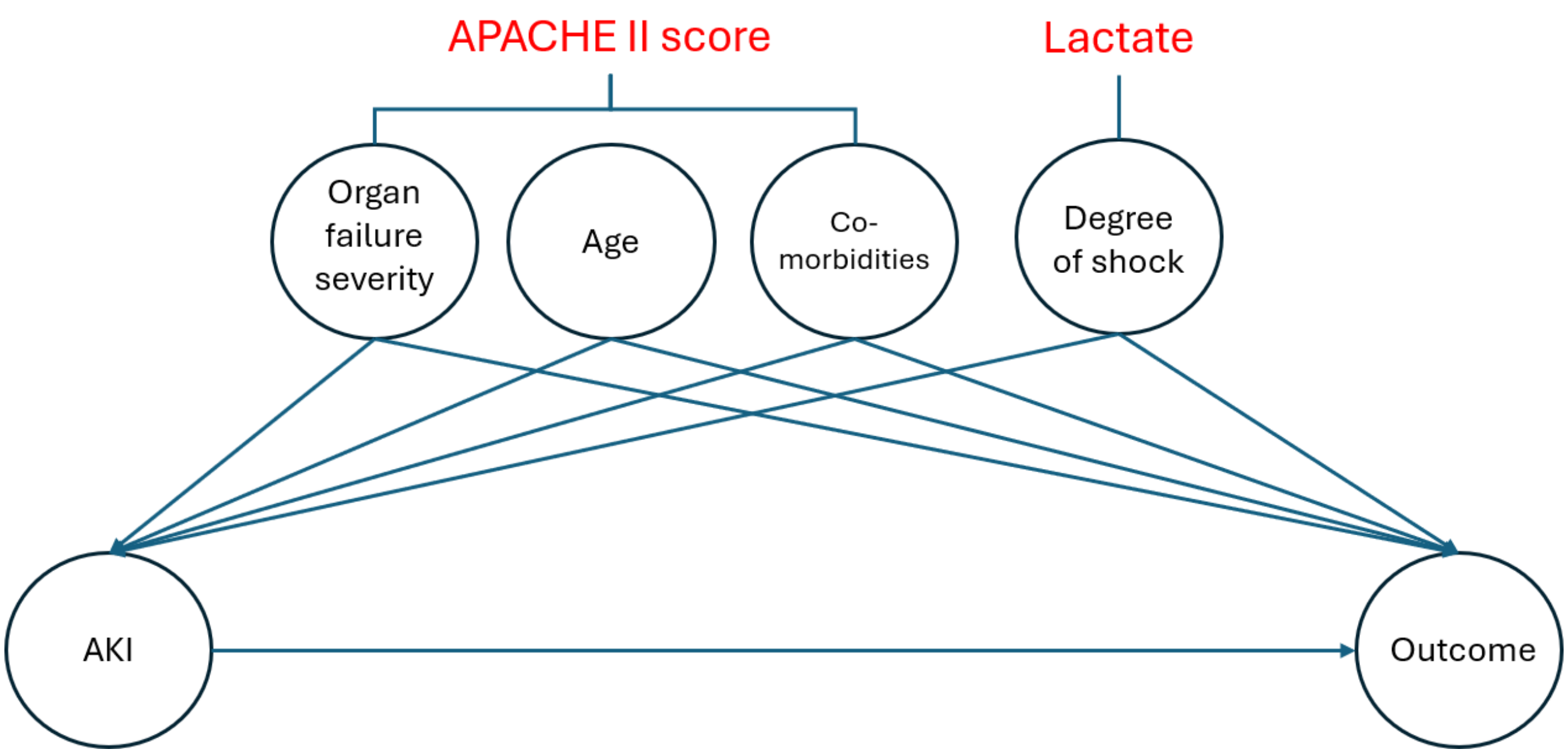

Supplementary Figure S2 Fow diagram showing the cohorts of patients used for each analysis

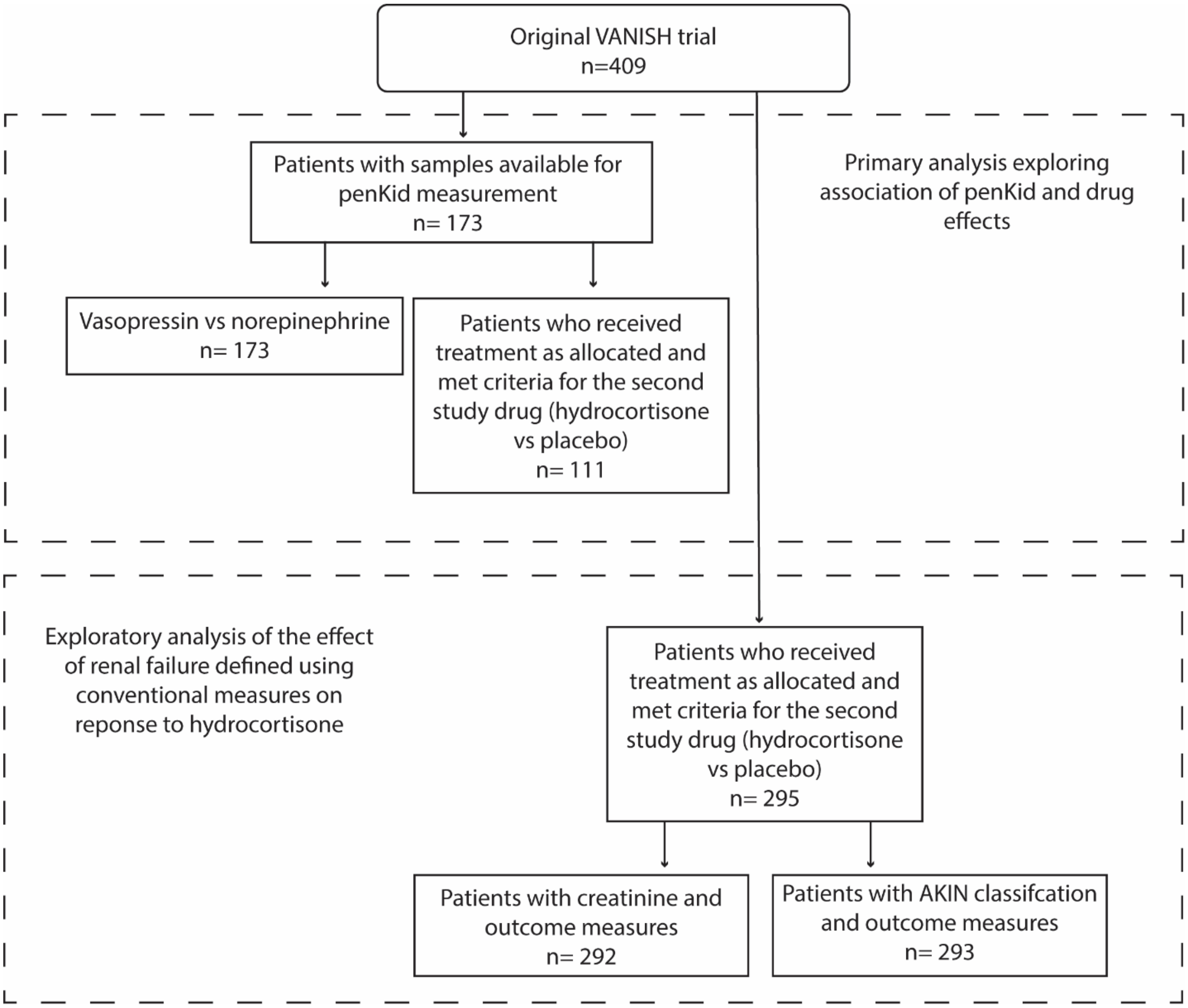

Supplementary Figure S3 Venn diagrams showing the overlap of AKI cohorts used to examine the effect of AKI on outcomes following the use of hydrocortisone. A. The 111 patients who had samples available for PENK measurement who received hydrocortisone or placebo comparing AKI defined by PENK, AKIN stage 3 or a serum creatinine 176.8 µmol/L. B. the 293 patients who revived hydrocortisone or placebo who had AKI classified by both AKIN and creatinine (1 patient had no creatinine value available). The numbers in the boxes outside of the circles represent those patients who did not have AKI under any definition.

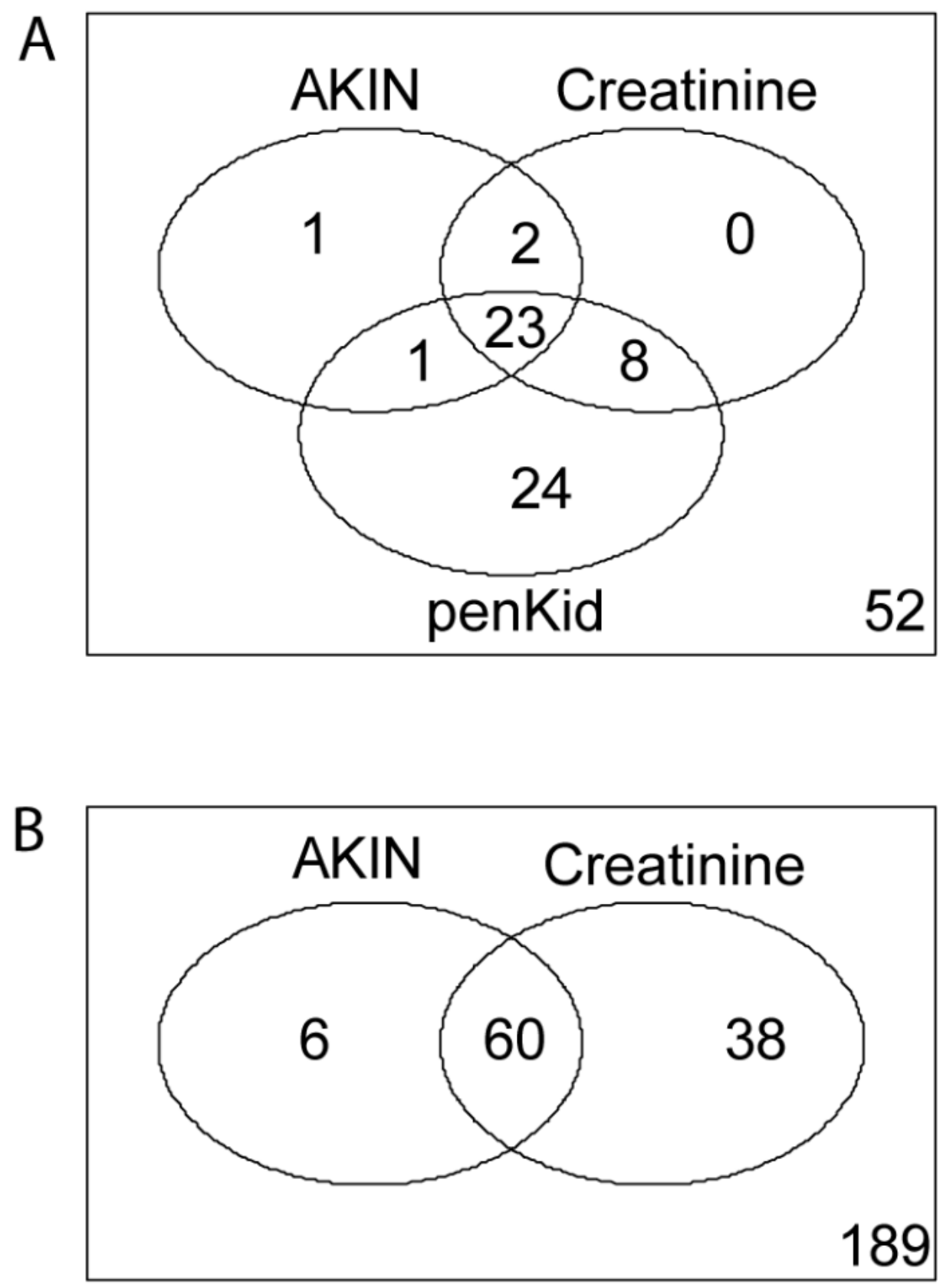

Supplementary Figure S4 Assessment of PENK (top row) and creatinine (bottom row) as continuous outcomes on A. kidney failure free days, B. kidney failure free days accounting for mortality as -1 days and C. 28-day mortality. The vertical dashed line represents thresholds used to dichotomize the patients: PENK of 89pg/ml in the top row and creatinine 176.8 µmol/L in the bottom row. Blue – placebo, orange – hydrocortisone. Where PENK was below the limit of detection (<29.9pg/ml, n=7) it was imputed as the limit.

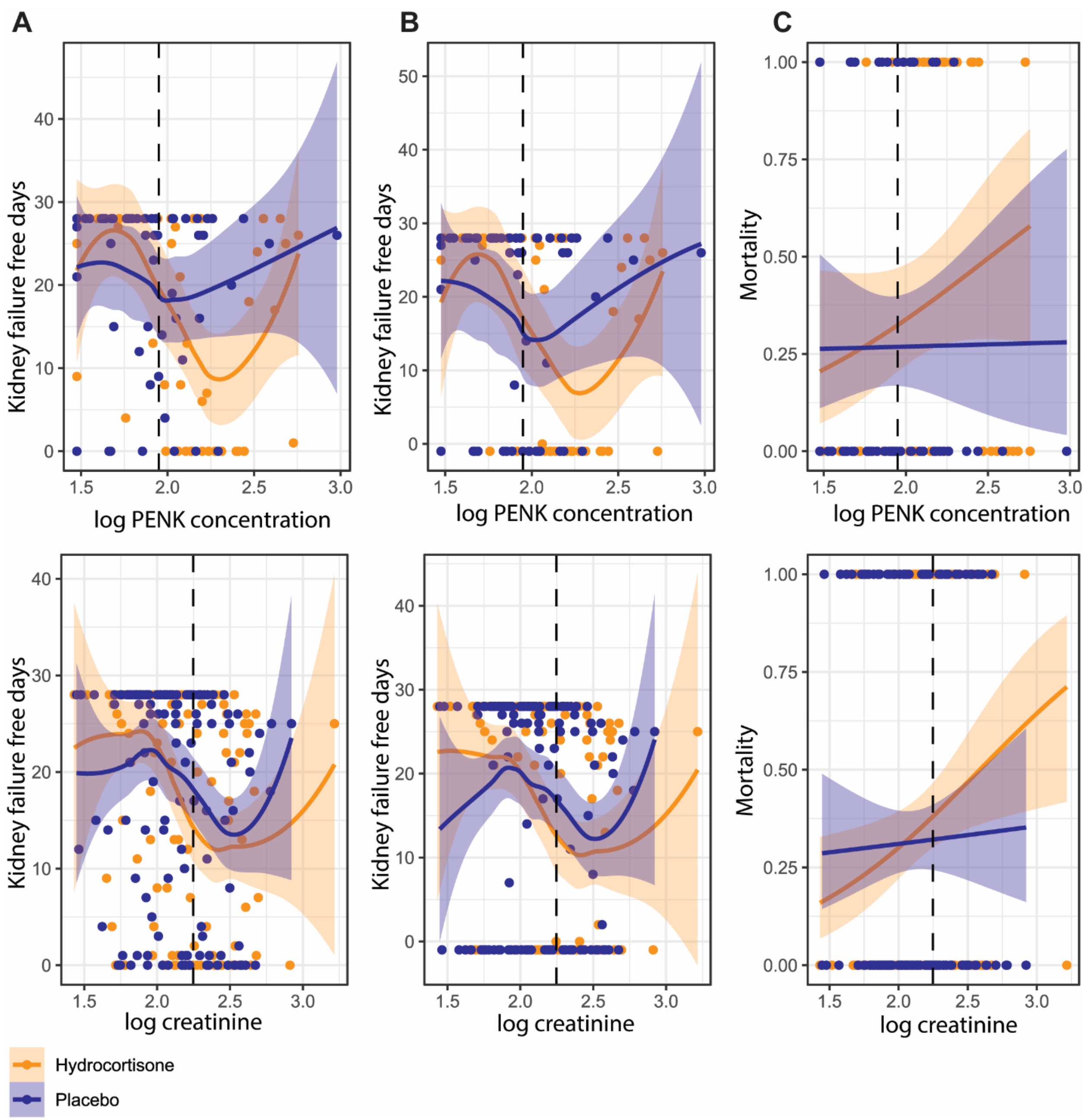

Supplement: Supplementary file 1 [file cc9-8-e1496-s001.pdf]
